# Vitamin D Deficiency Is Associated with Increased Osteocalcin Levels in Acute Aortic Dissection: A Pilot Study on Elderly Patients

**DOI:** 10.1155/2017/6412531

**Published:** 2017-07-02

**Authors:** Elena Vianello, Elena Dozio, Alessandra Barassi, Lorenza Tacchini, John Lamont, Santi Trimarchi, Massimiliano M. Marrocco-Trischitta, Massimiliano M. Corsi Romanelli

**Affiliations:** ^1^Department of Biomedical Sciences for Health, Università degli Studi di Milano, Via L. Mangiagalli 31, 20133 Milan, Italy; ^2^Laboratory of Molecular Pathology, I.R.C.C.S. Policlinico San Donato, Via R. Morandi 30, San Donato Milanese, 20097 Milan, Italy; ^3^Department of Health Sciences, Università degli Studi di Milano, Via A. Di Rudinì 8, 20142 Milan, Italy; ^4^Randox Laboratories Ltd., R&D, 55 Diamond Road, Crumlin, Antrim, Belfast BT29 4QY, UK; ^5^Thoracic Aortic Research Center, I.R.C.C.S. Policlinico San Donato, Piazza E. Malan 1, San Donato Milanese, 20097 Milan, Italy; ^6^Laboratory Medicine Operative Unit-1, Clinical Pathology, I.R.C.C.S. Policlinico San Donato, Piazza E. Malan 1, San Donato Milanese, 20097 Milan, Italy

## Abstract

An imbalance between degradation and reconstruction of the aortic wall is one of the leading causes of acute aortic dissection (AAD). Vitamin D seems an intriguing molecule to explore in the field of AAD since it improves endothelial function and protects smooth muscle cells from inflammation-induced remodeling, calcification, and loss of function, all events which are strongly related to the aging process. We quantified 25-hydroxy vitamin D, calcium, parathormone, bone alkaline phosphatase, and osteocalcin levels in 24 elderly AAD patients to identify a potential pathological implication of these molecules in AAD. Median 25-hydroxy vitamin D (10.75 ng/mL, 25th–75th percentiles: 6.86–19.23 ng/mL) and calcium levels (8.70 mg/dL, 25th–75th percentiles: 7.30–8.80 mg/dL) suggested hypovitaminosis D and a moderate hypocalcemia. Thirty-eight percent of AAD patients had severe (<10 ng/mL), 38% moderate (10–20 ng/mL), and 24% mild 25-hydroxy vitamin D deficiency (20–30 ng/mL). A significant inverse correlation was observed between 25OHD and osteocalcin levels. All the other molecules were unchanged. A condition of hypovitaminosis D associated to an increase in osteocalcin levels is present in AAD patients. The identification of these molecules as new factors involved in AAD may be helpful to identify individuals at high risk as well to study preventing strategies.

## 1. Introduction

Increasing data suggest that vitamin D, in addition to its role in bone metabolism, may exert important extraskeletal effects. Among different tissues and organs, the cardiovascular system has been regarded as one of the main targets of vitamin D actions. Epidemiological studies indicated that serum 25-hydroxy vitamin D (25OHD) levels are inversely associated with atherosclerosis, hypertension, coronary artery disease, peripheral artery disease, and stroke as well as with most of the main traditional cardiovascular risk factors [1–7]. On the other end, clinical trials on vitamin D supplementation failed to demonstrate a reduction in cardiovascular disease events leaving open the question whether hypovitaminosis D is an epiphenomenon rather than an etiological factor [8].

Recent studies suggested the existence of an inverse relationship between 25OHD levels and the presence and size of aortic aneurysms and thoracic aortic dilatations [9–11]. The underlying mechanisms explaining these associations are not fully understood but probably deal with processes which may be affected by hypovitaminosis D, such as wall vessel inflammation, endothelial dysfunction, and artery wall integrity. In fact, in this regard, the potential anti-inflammatory properties of vitamin D [12, 13] as well as its ability to improve endothelial function and to protect smooth muscle cells from inflammation-induced remodeling, calcification, loss of function, and senescence have been suggested in different studies [14–18].

An imbalance between degradation and reconstruction of the aortic wall is also one of the leading causes of acute aortic dissection (AAD). AAD is an acute event caused by the formation of a tear within the artery wall and the creation of a false lumen within aortic wall between the intima and the media. It is associated with high morbidity and mortality if undiagnosed or not properly treated and is mainly characterized by cystic medial necrosis, elastic layer degradation, smooth muscle cell apoptosis, and inflammation [19]. Any condition which may affect aortic wall integrity may thus promote AAD.

Today, no studies assessed the vitamin D status in AAD patients. Thus, our aim was to evaluate whether vitamin D and vitamin D-related bone markers, which have also been related to metabolic and cardiovascular diseases, namely, osteocalcin (OC), parathormone (PTH), and bone alkaline phosphatase (BAP) [20, 21], are altered in AAD, also according to dissection localization at the ascending or descending aorta. The identification of new factors and pathways potentially associated and involved in AAD may be finally helpful to identify individuals at high risk as well to study preventing strategies.

## 2. Materials and Methods

### 2.1. Patients

This is a retrospective investigation study of 44 consecutive AAD patients who attended the I.R.C.C.S. Policlinico San Donato from 2005 to 2012 and were included in the International Registry of Aortic Dissection (IRAD) [22], according to the inclusion and exclusion criteria expected by the register. The study was conducted with the approval of local Ethics Committee (ASL Milano 2, Reference number 1409), and informed consent was signed by each patient. To reduce the effect of potential confounding factors, the following exclusion criteria were further applied: the known presence of generalized bone diseases (including hyperparathyroidism, rheumatoid arthritis, and Cushing's syndrome), malignant disease, recent major abdominal surgery, renal and liver diseases, Marfan syndrome or other genetic disorders, lack of clinical and biochemical data, and insufficient blood samples. Thus, a total of 24 patients were finally studied. According to Stanford classification, 19 were type A patients, having a dissection in the ascending aorta (proximal dissection), and 5 were type B, showing a dissection limited to the descending aorta (distal dissection).

AAD were repaired using deep or moderate hypothermic circulatory arrest and selective antegrade cerebral perfusion. All patients were managed with open distal anastomosis. Height, weight, medication intake, and risk factors were recorded. Body mass index (BMI, kg/m^2^) was calculated.

### 2.2. Biochemical Assays

Serum sample was obtained after centrifugation at 1000*g* for 15 min and then immediately stored at −20°C until analysis. Serum 25OHD levels were measured by a chemiluminescence assay (Total 25OHD assay, DiaSorin, Saluggia, Italy). The lower detection limit was 4 ng/mL; the intra- and interassay coefficients of variation (CVs) were 3.7 to 7.7% and 5.8 to 10.9%, respectively. BAP was quantified by a direct, 2-site sandwich-type immunoluminometric assay utilizing two monoclonal antibodies (BAP OSTASE, DiaSorin). The lower detection limit was 1.5 μg/L; the intra- and interassay CVs were 3.2 to 4.0% and 6.5 to 8.1%, respectively. A direct, 2-site, sandwich-type immunoluminometric test which utilizes directly coated microparticles was used for OC detection (Osteocalcin, DiaSorin). The lower detection limit was 0.5 ng/mL; the intra- and interassay CVs were 3.0 to 8.0% and 4.0 to 9.0%, respectively. 1–84 PTH was quantified with a 2-site, sandwich-type immunoluminometric test using directly coated microparticles (1–84 PTH assay, DiaSorin). The lower detection limit was 4.0 pg/mL; the intra- and interassay CVs were 3.0 to 5.9% and 5.5 to 9.0%, respectively.

All analyses were performed using the LIAISON Analyzer (DiaSorin). Calcium was quantified by a colorimetric method using Vitros 5600 System (Ortho Clinical Diagnostics, Rochester). The lower detection limit was 4.0 mg/dL; the intra- and interassay CVs were 0.04 to 0.12% and 0.9 to 1.9%, respectively.

### 2.3. Statistical Analysis

The normality of data distribution was assessed by the Kolmogrov-Smirnoff test. Data were expressed as mean ± standard deviation (SD), median (25th–75th percentiles), or number and percentage. *T*-test, for normal variables, or Mann–Whitney *U*-test, for nonparametric variables, was used to compare two groups. Chi-square test was used for categorical outcomes. For multiple comparison (three groups), the Bonferroni correction was used. Pearson (for normal distributed data) or Spearman (for non-normal distributed data) correlation tests were used to test the univariate association between variables. All statistical analyses were performed using GraphPad Prism 5.0 biochemical statistical package (GraphPad Software, San Diego, CA). A *p* value <0.05 was considered significant.

## 3. Results and Discussion

### 3.1. Results

The demographic, anthropometric, and clinical characteristics of patients enrolled in the study are reported in Table 1. In AAD patients, the median serum 25OHD levels (10.75 ng/mL) suggest an overall condition of hypovitaminosis D. None of the patients had 25OHD levels higher than 30 ng/mL, the threshold for optimal vitamin D status. The levels of 25OHD < 10 ng/mL, 10–20 ng/mL, and 20–30 ng/mL were designated as severe deficiency, moderate, and mild deficiency, respectively. According to this classification, the 38% of AAD patients had severe 25OHD deficiency, 38% moderate 25OHD deficiency, and 24% mild 25OHD deficiency.

Relative to the other vitamin D-related bone turnover markers, OC levels (17.95 ± 8.1 ng/mL) as well as BAP (7.39 ± 3.2 mg/L) and PTH (24.40, 14.10–39.68 pg/mL) were within the ranges of normality. After AAD patient classification according to 25OHD status, we observed a significant increase in OC concentrations (*p* < 0.05 for 25OHD < 10 ng/mL versus 25OHD > 20 ng/mL) at decreasing 25OHD level.

**Figure 1:**
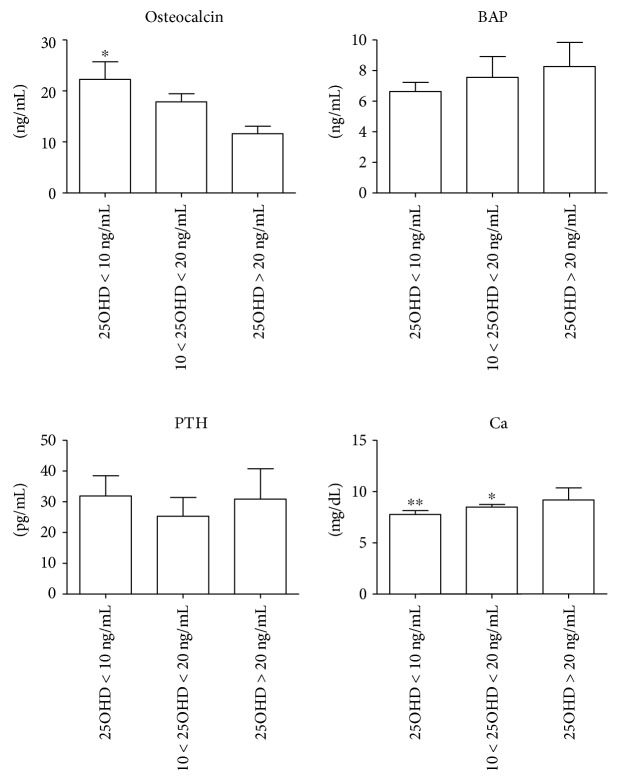
Evaluation of osteocalcin, bone alkaline phosphatase (BAP), parathormone (PTH), and calcium levels in patients with acute aortic dissection (AAD) according to plasma 25OHD concentration. AAD patients were stratified into three groups (severe deficiency: 25OHD < 10 ng/mL; moderate deficiency: 10–20 ng/mL; mild deficiency: >20 ng/mL) according to plasma 25OHD concentration. ^∗^*p* < 0.05 and ^∗∗^*p* < 0.01 versus 25OHD > 20 ng/mL.

Differently, statistically significant differences were observed neither in BAP and PTH levels among groups (Figure 1) nor in any other clinical or demographic parameters studied (Table 1). The vitamin D status of AAD patients was further evaluated with regard to cut-off value of 15 ng/mL, which has been suggested as a more accurate limit below which cardiometabolic dysfunction may be observed [23–25]. In patients with 25OHD levels lower than 15 ng/mL, OC was higher (18.60, 17.13–23.38) compared to the other group (14.80 ± 4.52, *p* < 0.05), whereas no differences were observed in the levels of all the other markers (data not shown). Univariate correlation analysis clearly showed the existence of an inverse association between 25OHD and OC (*r* = −0.610, *p* = 0.016). No correlation was observed with the other parameters, instead.

Mean calcium levels suggested a mild condition of hypocalcemia in AAD patients. As expected, calcium levels decreased at decreasing 25OHD levels (*p* < 0.01 for 25OHD < 10 ng/mL versus 25OHD > 20 ng/mL; *p* < 0.05 for 10 ng/mL < 25OHD < 20 ng/mL versus 25OHD > 20 ng/mL). Anyway, univariate correlation analyses with 25OHD and the other parameters of calcium metabolism did not reach the statistical significance (25OHD: *r* = −0.266, *p* = 0.245; OC: *r* = −0.126, *p* = 0.586; BAP: *r* = −0.041, *p* = 0.859; PTH: *r* = −0.049, *p* = 0.834).

**Figure 2:**
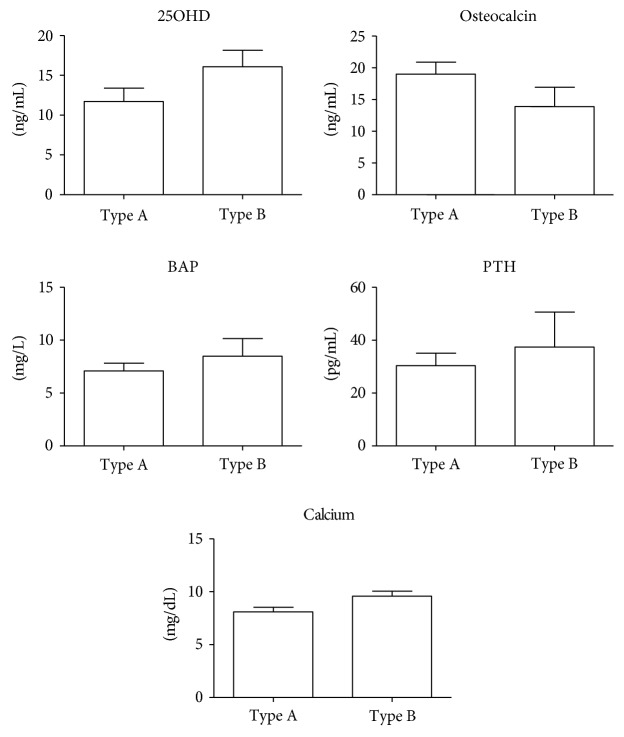
Evaluation of 25OHD, osteocalcin, bone alkaline phosphatase (BAP), parathormone (PTH), and calcium levels in patients with acute aortic dissection (AAD) classified according to dissection localization: types A and type B.

AAD patients were further classified according to dissection localization. Levels of 25OHD and OC were, respectively, higher and lower in type B compared to type A patients, although just over the limit of statistical significance (Figure 2). None of type B patients displayed 25OHD severe deficiency. BAP and PTH levels were almost the same in the two groups, as well as calcium.

## 4. Discussion

The main observation of our study is that AAD patients displayed hypovitaminosis D and there was an inverse relationship between 25OHD and OC levels. Otherwise, no changes in the concentrations of the other bone-related molecules, according to vitamin D status, have been observed in these patients.

Adequate vitamin D levels are first of all important for calcium absorption and thus the regulation of calcium homeostasis. Since low concentration of 25OHD is one of the main cause of hypocalcemia, it is not surprising to observe a trend of decrease in calcium concentration according to the vitamin D status of AAD patients. Otherwise, we did not observe an increase in PTH secretion as a compensatory mechanism to keep calcium concentration normal, as well as any significant correlation between calcium and vitamin D levels. Since PTH secretion is promoted by a decrease in free calcium level, it is possible that, despite the decrease in total calcium level, free calcium did not change. Unfortunately, in the present study, we could measure total but not free calcium.

The unchanged levels of the bone turnover marker BAP seemed also to suggest the lack of activation of bone-related compensatory mechanisms in the regulation of calcium homeostasis in AAD patients. BAP is mainly produced by ostoblasts and plays important roles in bone matrix calcification [26]. Increased BAP levels are indicative of a higher osteoblastic activity as a compensatory mechanism towards increased osteoclastic activities, as observed, in osteomalacia, a condition characterized by a slight increase in BAP levels which are usually normalized after vitamin D treatment [27]. Due to vitamin D deficiency, one could thus expect to observe the activation of bone pathways promoting bone-calcium resorption but this seems not to be the main consequence of hypovitaminosis D in AAD patients.

In the present study, we also evaluated another bone matrix protein, OC, a vitamin D-related product which is released by osteoblasts and has potential role not only in osteoblast-osteoclast interaction and bone resorption but also in metabolism. Concerning OC, we observed the existence of an inverse correlation between this molecule and vitamin D. The fact that we could not observe any association between hypovitaminosis D and bone turnover did not exclude the possibility that reduced vitamin D levels may have consequences on vascular and cardiovascular health. Patients evaluated in this study, in fact, displayed an acute vascular event which was the result of inflammation-related processes and alteration in the composition of the media tunica of the vascular wall. Recent reports indicated that OC is associated to arterial diseases, thus being a molecule able to exert important effects also on vascular tissues which is similar to bone tissue in term of regulation of mineral homeostatsis [21]. In addition to OC, another molecule mainly produced by the bone, osteopontin, emerged as a novel player in arterial remodeling, inflammation, and the promotion of fibrosis, thus suggesting the same ability of these bone-derived molecules to target the cardiovascular system [28, 29]. Previous studies indicated a strong association between hypovitaminosis D and the presence and extend of arterial diseases, regardless of traditional cardiovascular risk factors and different cardiovascular pathologies, from coronary artery disease, to peripheral artery disease and aneurysms [1–7]. The association with the presence and the extent of aneurysms has also been observed regardless of atherosclerosis which is considered one leading cause of aneurysm formation, thus suggesting a potential direct effect of vitamin D action on artery walls [9–11]. In this regard, vitamin D receptor is expressed both on endothelial as well as on smooth muscle cells where vitamin D can control cellular survival and local inflammation by preventing macrophage infiltration and the consequent production of proinflammatory and detrimental mediators [30, 31]. This means that one of the main vitamin D roles at vascular level may be its ability to play as an anti-inflammatory agent. Reduced vitamin D levels have also been associated to increased arterial stiffness and vascular calcification [32]. Since endothelial dysfunction, inflammation, and degeneration of the tunica media are key event not only in simple aneurysm formation but also in AAD, we found it interesting to explore vitamin D and OC levels in AAD. Our study did not describe the molecular mechanisms that could link hypovitaminosis D to vascular degeneration and AAD but indicated that both vitamin D and OC may play an important pathogenetic role which needs to be further explored, anyway. According to our data, variation in OC levels related to that of vitamin D seemed to reflect the activation of local mechanisms at vascular level rather than an alteration in bone metabolism. In fact, this inverse correlation between vitamin D and OC without changes in the levels of other bone-related molecules strongly reinforced the role of OC as an “extrabone” molecule. OC is mainly produce by osteoblasts where its synthesis is just regulated by vitamin D and where it plays important activities in bone matrix mineralization. OC exists in two main form: carboxylated, promoted by vitamin K, and undercarboxylated. In this latter form, it has less affinities towards calcium and hydroxyapatite and it is easily secreted and plays important endocrine activities, such as the stimulation of insulin secretion, the increase in insulin sensitivity, and the reduction of blood glucose levels [21]. Both forms are anyway secreted. The quantification of total OC, as done in our study, is an overall index of both bone- and nonbone-related effects. We also know that OC may be produced by endothelial progenitor cells [33] which may differentiate into OC-producing cells with a potential involvement in vascular dysfunction and in the pathogenesis of AAD. Also, the vascular smooth muscle cells can differentiate into osteoblast-like cells [34, 35].

This has been mainly observed in the presence of inflammation [36] that, as suggested in different previous papers, may be promoted by hypovitaminosis D [12, 37, 38]. To better define the potential pathogenetic role of OC in AAD, it would be interesting to explore on aortic tissues from AAD patients the relationship among OC expression and vitamin D and its receptor.

In our study, we also compared the type of AAD according to dissection localization. Usually, type A AAD, that is, at ascending aorta, is associated to worsen prognosis. By comparing the two types, we could not observe any difference in the levels of the evaluated molecules, although in type B patients, levels of 25OHD were higher and OC lower compared to type A, but at the limit of the statistical significance, probably due to the reduced number of type B patients.

## 5. Conclusions

Our study suggests that in AAD hypovitaminosis D is not associated to changes in bone-related metabolic pathways but is inversely related to OC which could be an interesting molecule able to mediate the effect of inadequate 25OHD level at vascular level. Future studies exploring local vascular mechanisms of vitamin D and OC could be helpful to better highlight the role of these molecules in this disease. Whether maintaining an optimal vitamin D status could be a preventing strategy needs also further analyses.

## Figures and Tables

**Table 1:** Characteristics of participants included in the study.

	AAD patients	25OHD < 10 ng/mL	10 ng/mL < 25OHD < 20 ng/mL	25OHD > 20 ng/mL
	(*n* = 24)	(*n* = 9)	(*n* = 9)	(*n* = 6)
Age (years)	62.67 ± 12.11	58.00 ± 14.21	61.11 ± 8.34	72.00 ± 9.59
Gender				
Male (*n*, %)	15, 62.5	7, 77.78	8, 88.89	4, 66.66
Female (*n*, %)	9, 37.5	2, 22.22	1, 11.11	2, 33.33
BMI (kg/m^2^)	23.09 ± 6.09	23.23 ± 9.77	23.57 ± 2.68	25.28 ± 3.88
Type of dissection			
A	19, 79.17	9, 100.00	6, 66.67	4, 66.67
B	5, 20.83	0, 0.00	3, 33.33	2, 33.33
First SBP	130.00 ± 50.88	117.22 ± 27.05	141.22 ± 70.15	79.00 ± 27.02
First DBP	60.00 (60.00–92.50)	66.00 (55.00–80.00)	83.75 ± 38.15	60.00 (60.00–92.50)
Risk factors (*n*, %)				
Hypertension	18, 75.0	6, 66.67	6, 66.67	6, 100.00
Diabetes	—	—	—	—
Atherosclerosis	6, 25.0	2, 22.22	1, 11.11	3, 50.00
Known aortic aneurism	2, 6.3	1, 11.11	1, 11.11	0, 0.00
Prior aortic dissection	—	—	—	—
Mitral valve disease	1, 4.2	0, 0.00	1, 11.11	0, 0.00
Bicuspid aortic valve disease	3, 12.5	3, 33.33	0, 0.00	0, 0.00
Aortic valve disease	1, 4.2	1, 11.11	0, 0.00	0, 0.00
Tricuspid valve disease	—	—	—	—
Peripartum state	—	—	—	—
Other aortic disease	—	—	—	—
Cocaine abuse	1, 4.2	1, 11.11	0, 0.00	0, 0.00
Smoking	6, 25.0	3, 33.33	1, 11.11	2, 33.33
ACE inhibitors	4, 16.7	1, 11.11	2, 22.22	1, 16.67
ARB	1, 4.2	0, 0.00	0, 0.00	1, 16.67
Beta blockers	13, 54.2	6, 66.67	4, 44.44	3, 50.00
Ca-channel blockers	4, 16.7	1, 11.11	2, 22.22	1, 16.67
Diuretic	3, 12.5	0, 0.00	1, 11.11	2, 33.33
Nitroprusside	6, 25.0	0, 0.00	4, 44.44	2, 33.33
Other vasodilators	7, 29.2	4, 44.44	2, 22.22	1, 16.67
Vasopressors	1, 4.2	0, 0.00	0, 0.00	1, 16.67
25OHD (ng/mL)	10.75 (6.86–19.23)	6.29 ± 1.64^a,b^	10.60 (11.00–13.60)^b^	23.02 ± 2.55
Osteocalcin (ng/mL)	17.95 ± 8.14	22.26 ± 10.39^c^	17.86 ± 4.85	11.62 ± 3.57
BAP (mg/L)	7.39 ± 3.24	6.33 ± 1.78	7.56 ± 4.06	8.27 ± 3.84
PTH (pg/mL)	24.40 (14.10–39.68)	31.91 ± 19.65	25.32 ± 18.22	30.88 ± 22.09
Ca (mg/dL)	8.70 (7.30–8.80)	7.77 ± 0.99^d^	8.70 (8.25–8.78)^c^	10.75 ± 2.03

Data are expressed as mean ± SD, median (25th–75th percentiles), or number and proportions. BMI: body mass index; NW: normal weight; OW: overweight; OB: obese; SBP: systolic blood pressure; DBP: diastolic blood pressure; ACE: angiotensin-converting enzyme. ARB: angiotensin II receptor blocker; 25OHD: 25-hydroxy vitamin D; BAP: bone-specific alkaline phosphate protein; PTH: 1–84 parathormone; Ca: calcium. ^a^*p* < 0.001 versus 10 ng/mL < 25OHD< 20 ng/mL; ^b^*p* < 0.001 versus 25OHD > 20 ng/mL; ^c^*p* < 0.05 versus 25OHD > 20 ng/mL; ^d^*p* < 0.01 versus 25OHD > 20 ng/mL.
